# Physical Activity and Quality of Life in Retinitis Pigmentosa

**DOI:** 10.1155/2017/6950642

**Published:** 2017-05-17

**Authors:** Joshua D. Levinson, Ethan Joseph, Laura A. Ward, Joe R. Nocera, Machelle T. Pardue, Beau B. Bruce, Jiong Yan

**Affiliations:** ^1^Department of Ophthalmology, Emory University School of Medicine, Atlanta, GA, USA; ^2^Rollins School of Public Health, Emory University, Atlanta, GA, USA; ^3^Center for Visual and Neurocognitive Rehabilitation, Atlanta VA Medical Center, Decatur, GA, USA; ^4^Department of Neurology, Emory University School of Medicine, Atlanta, GA, USA; ^5^Department of Biomedical Engineering, Georgia Institute of Technology, Atlanta, GA, USA

## Abstract

**Purpose:**

Aerobic exercise has been found to be neuroprotective in animal models of retinal degeneration. This study aims to report physical activity levels in patients with RP and investigate the relationship between physical activity and vision-related quality-of-life (QOL).

**Materials and Methods:**

A retrospective study of adult patients with RP examined in 2005–2014. Physical activity levels were assessed using the Godin Exercise Questionnaire. The NEI-Visual Function Questionaire-25 (VFQ-25), SF-36 General Health survey, and Pepper Assessment Tool for Disability (PAT-D) were administered.

**Results:**

143 patients participated. 81 (56.6%) patients were classified as “active” and 62 (43.4%) as “insufficiently active” by Godin score. VFQ-25 revealed statistically significant differences between the active and insufficiently active patients, including overall visual function (53.3 versus 45.1, *p* = 0.010), color vision (73.8 versus 52.9, *p* < 0.001), and peripheral vision (34.3 versus 23.8, *p* = 0.021). The physical component of the SF-36 and the PAT-D survey also demonstrated statistically significant differences (47.2 versus 52.9, *p* = 0.002; 24.3 versus 30.0, *p* = 0.010). Active patients had a higher initial Goldmann visual field (GVF) score (74.8 versus 60.1 degrees, *p* = 0.255) and final GVF score (78.7 versus 47.1 degrees, *p* = 0.069) but did not reach statistical significance.

**Conclusions:**

In RP, increased physical activity is associated with greater self-reported visual function and QOL.

## 1. Introduction

Retinitis pigmentosa (RP) is an inherited retinal dystrophy affecting more than 1 million individuals worldwide [1]. Early symptoms include night blindness and peripheral visual field constriction. Advanced disease is characterized by a small central tunnel visual field that may progress to loss of central vision. In a small number of patients, RP leads to total blindness. Currently, there are no proven treatments to slow the progression of RP. Given the lack of significant treatment options, anticipated vision loss in RP can be emotionally devastating [2].

Exercise is well known to have a positive impact on both physical and psychological health. Exercise has even been shown to enhance memory and promote hippocampal neuroregeneration [3] suggesting a neuroprotective effect. Previous studies have suggested a beneficial effect of exercise on common ocular conditions such as age-related macular degeneration (AMD) [4, 5], glaucoma [6], and cataract [7]. Vision loss in RP is due to progressive loss of rod and cone photoreceptors [1]. Recently, exercise has been shown to have neuroprotective effects on photoreceptors in mouse models of retinal degeneration [8, 9].

While many studies have investigated exercise and its impact on the health of the human body, the effect of exercise on the progression of retinal degeneration in human subjects is poorly understood. This retrospective case-control study will attempt to report baseline physical activity levels in individuals with RP and to investigate the relationship between physical activity and visual function reported from three quality of life (QOL) surveys.

## 2. Methods

A retrospective review of the medical record was performed for adult patients with retinitis pigmentosa evaluated at the Emory Eye Center between October 2004 and April 2015. Subjects were identified through a clinical database of patients with retinal degenerative conditions. This study was approved by Emory University's Institutional Review Board and adhered to the tenets of the Declaration of Helsinki.

Clinical data was obtained from the medical record and included age, gender, race, and medical history. Goldmann visual fields (GVF) were reviewed. Visual field scores were obtained for each eye by summation of the degree of preserved visual field across the horizontal and vertical meridians using the III4e isopter. As quality of life of a patient is most affected by vision in the better-seeing eye, the eye with the larger initial visual field was analyzed for statistical purposes. For subjects with multiple visual fields, data was collected for the first and last tests within the study period. When available, electroretinograms (ERGs) were reviewed from each patient's initial clinic visit. The electrophysiologist reported photoreceptor function as “normal” or “mild”, “moderate,” or “severe” photoreceptor dysfunction. As many ERG responses were indistinguishable from noise due to the advanced nature of the disease, a decision was made not to include quantitative ERG measurements. The clinical-intake history form asked patients if they have diabetes, hypertension, or cardiovascular disease. Patients who reported a history of one or more of these conditions were classified as having “vascular disease.”

Subjects were contacted by telephone and four validated questionnaires were administered to those that consented. Additional questions including smoking history and employment status were asked at the end of the survey.

### 2.1. National Eye Institute Visual Function Questionnaire-25 (NEI VFQ-25)

The NEI VFQ-25 was administered to evaluate subjective visual function and vision-related quality of life [10, 11]. NEI VFQ-25 consists of 25 questions designed to assess 12 aspects of daily living: general health, general vision, near vision, distance vision, driving, peripheral vision, color vision, ocular pain, role limitation, dependency, social function, and mental health.

### 2.2. Pepper Assessment Tool for Disability (PAT-D)

The PAT-D is a 19-item questionnaire designed to assess mobility, activities of daily living (ADL), and instrumental activities of daily living (IADL) [12, 13]. Participants rate their level of difficulty performing each activity on a 5-point Likert scale ranging from 1 (“no difficulty”) to 5 (“unable to do”).

### 2.3. 36-Item Short Form Health Survey (SF-36)

General health-related quality of life was assessed with the SF-36 survey [14]. The SF-36 consists of 8 subscores: physical functioning, role limitations due to physical health problems, role limitations due to emotional health problems, social functioning, freedom from pain, energy or fatigue, emotional well-being, and general health perceptions. Scores range from 0 (maximum impairment) to 100 (no impairment). Subscores are used to calculate a Physical Component Summary (PCS) and Mental Component Summary (MCS).

### 2.4. Godin Leisure-Time Exercise Questionnaire (GLTEQ)

An excerpt from the Godin Leisure-Time Exercise Questionnaire [15] was used to determine the frequency and intensity of physical activity. Respondents report the number of times per week that they engage in mild, moderate, or strenuous physical activity which is then multiplied by 3, 5, or 9 metabolic equivalents, respectively, and summated into a total score. Subjects were classified as either “active” or “insufficiently active” using a cutoff score of 24 as suggested by prior studies [16]. While some studies in the literature exclude mild physical activity [16], we elected to include mild physical activity in our calculation with the understanding that RP patients with advanced vision loss may have difficulty performing more strenuous physical activity.

### 2.5. Statistical Methods

Descriptive demographics and medical history statistics were calculated for the full cohort. Quality of life measures were calculated overall and by Godin activity score category (active versus insufficiently active). The differences between the activity levels were tested using a two-sample *t*-test for the overall quality of life scores, as well as the general vision, color vision, and peripheral vision subscores of the VFQ-25 and the physical functioning, social function, and general health subscores of the SF-36.

Two-sample *t*-tests were also used to examine the differences between the summation of the horizontal and vertical GVF III4e measurements in the better eye by activity level. This was done for both the initial and final measurements, as well as the difference between the initial and final GVF III4e measurements. The summed GVF III4e measurements were also examined with two-sample *t*-tests by current smoking status and presence of vascular disease. These relationships were also tested using a linear regression model, controlling for age.

All statistical tests were performed using SAS v9.4 software and tested using an alpha of 0.05.

## 3. Results

143 patients completed the telephone survey out of 496 patients in the clinical database (28.8%). The mean age of study participants was 46.9 years. Demographic and medical history data is presented in Table 1.

The average Godin exercise score was 32.6. Eighty-one patients (56.6%) were classified as “active” while 62 (43.4%) were “insufficiently active.” Active patients were found to have a higher overall NEI VFQ-25 score than the insufficiently active patients (53.3 versus 45.1, *p* = 0.010) (Table 2). General vision, color vision, and peripheral vision were identified as subscores of particular interest. Increased physical activity was associated with higher subscores in peripheral vision (*p* = 0.021) and color vision (*p* < 0.001).

In evaluation of general health-related quality of life, active patients scored significantly higher on the physical component summary score of the SF-36 (52.9 versus 47.2, *p* = 0.002) but no significant difference was seen for the mental component summary (51.1 versus 51.7, *p* = 0.731). Active patients were found to have significantly less disabilities on the PAT-D survey (24.34 versus 30.0, *p* = 0.010).

Initial visual field measurements were available for 120 patients. The mean initial GVF summation score was 67.9 degrees. The relationship between GVF scores and activity level is presented in Table 3. Active subjects had a higher initial GVF score than the insufficiently active subjects (Table 3), but this difference did not reach statistical significance (74.8 versus 60.1, *p* = 0.255). Follow-up visual fields were available for 52 patients. In this cohort, the initial mean GVF score was 90.2 degrees in the active group and 55.3 degrees in the insufficiently active group. The final GVF scores were also higher in the active group, and this difference approached but did not reach statistical significance (78.7 versus 47.1 degrees, *p* = 0.069). For both active and insufficiently active patients, the mean GVF score decreased over the follow-up period (−11.54 degrees active versus −6.62 degrees insufficiently active, *p* = 0.6987).

The association between age and physical activity level was found to approach statistical significance, with increased physical activity seen in younger patients (*p* = 0.056). However, age was not found to be associated with degrees of preserved initial visual field in a univariate analysis (*p* = 0.1948) or in a multivariate analysis with activity level as a covariate (*p* = 0.8456).

Sixty-seven percent of respondents reported a history of tobacco use. No significant difference in GVF scores was seen between smokers and nonsmokers (65.7 versus 72.2, *p* = 0.63) (Table 4). However, patients with a history of vascular disease had significantly more constricted visual fields than patients without vascular disease (46.2 versus 80.0, *p* = 0.011). Vascular disease remained associated with a reduced visual field even when controlling for age (*p* = 0.032).

Sixty-six patients reported current employment out of 142 respondents (46.5%). Current employment was associated with more favorable scores on the PAT-D, VFQ-25, and the Physical Component Summary of the SF-36 (Table 5). No association was seen between employment and the Mental Component Summary.

## 4. Discussion

In this survey of 143 patients with RP, approximately 4 in 10 patients were found to have “insufficiently active” lifestyles. The mean GLTEQ score of 32.6 reveals that patients with RP have a physical activity level that falls between previously published values for patients with physical disabilities [17] and healthy controls [18]. The high rate of physically inactivity seen in our study is consistent with the findings of An et al. which reported a significantly higher rate of physical inactivity in RP patients compared to healthy controls (50.8% versus 27.3%) [19].

This study revealed significantly better self-reported overall visual function, color vision, and peripheral vision in active patients as measured by the NEI VFQ-25. Visual field scores were higher for active patients at both the initial and final measurement, although these differences did not reach statistical significance. Quantifying visual function in RP patients is difficult. Central visual acuity is often preserved until significant visual field loss has occurred. In our study, ERGs demonstrated severe photoreceptor dysfunction in 73.2% of patients on presentation. ERG measurements in these patients are often indistinguishable from noise which makes them difficult to follow over time. Goldmann visual fields are often relied on to track visual loss, but the sensitivity is limited. GVFs have increased variability in RP patients [20] and this variability increases with disease severity [21]. The use of a survey, like the NEI VFQ-25, provides a subjective means to evaluate visual function and should be included as a focus of future prospective studies evaluating the effects of exercise on RP.

Pardue and colleagues found aerobic exercise to have a neuroprotective effect on photoreceptors in mouse models of retinal degeneration which they demonstrated was mediated through increases in brain-derived neurotrophic factor (BDNF) [8, 9]. In humans, BDNF is one of the principle growth factors known to mediate the effects of exercise on the brain [22]. Further research is warranted to investigate if BDNF may have a protective effect on photoreceptors in humans.

While little is known regarding the effect of exercise on human retinal degeneration, several studies have demonstrated a protective effect of aerobic exercise in other neurodegenerative diseases. In a large meta-analysis, Beckett et al. demonstrated that physical activity is associated with a reduced risk of Alzheimer's disease in adults over the age of 65 [23]. Hernández et al. performed a systematic review of the literature between 2003 and 2013 [24]. They found that exercise may improve cognition and performance of daily activities in patients with Alzheimer's disease. Exercise has also been reported to be beneficial in delaying the onset of Parkinson's disease and Huntington's disease [22].

Less than half of the adult patients included in this survey reported current employment. Not surprisingly, patients who were employed scored more favorably on QOL and disability surveys. This high level of unemployment is indicative of the magnitude of disability seen in patients with advanced RP, a problem that is only compounded in families with multiple affected members.

Two-thirds of patients reported a history of cigarette smoking. It is unclear why such a high rate of smoking was found although the wording of the questionnaire, which included both current and former smoking, may influence it. A case-control study from Korea found a significantly lower rate of current smokers among RP patients compared to healthy controls [19]. It is notable that cigarette smoking was not found to be associated with lower visual field scores in our study. Smoking promotes oxidative stress, and smoking cessation is typically recommended for patients with retinal diseases.

In our study, vascular disease was found to be a significant risk factor for visual field loss. While retinal vascular attenuation in RP has traditionally been thought to occur secondary to neuronal cell loss, a role for impaired ocular blood flow in the pathogenesis of RP has been suggested [25].

This study is limited by its retrospective nature as well as several other shortcomings. The survey response rate of 28.8% is relatively low, although almost all patients who were successfully contacted chose to participate in the survey. The majority of nonresponders were patients who could not be located due to out-of-date contact information in the medical record. RP is a rare disease and most studies for RP are limited by sample size. While our study includes a relatively large sample size, only a small number of patients have follow-up visual fields for comparison. This small number may have limited our ability to reach statistical significance in the visual field analysis. Furthermore, GVF scores decreased over time in both groups. This indicates that increased physical activity does not completely halt progression of disease. Further studies are warranted to better quantify the effect of exercise on the rate of disease progression. In this study, frequency and duration of physical activity were assessed at a single point in time. However, photoreceptor degeneration may begin early in life even in late onset symptomatic patients [1, 26]. Lifelong habits of exercise would be a better indicator of a patient's exercise pattern rather than an assessment from any single point. Age was not found to be significantly associated with visual field scores, although the association between age and physical activity level approached borderline significance. Given that visual field loss in RP is progressive with age, we recommend controlling for age in further prospective evaluations of exercise in RP.

We aimed to report baseline exercise patterns in patients with RP and investigate the relationship between physical activity and visual function reported from three quality of life (QOL) surveys. To our knowledge, this study represents the first clinical investigation of exercise in RP. We have demonstrated that higher physical activity levels are associated with greater self-reported visual function, as measured by the NEI VFQ-25, and lower levels of disability. Due to the limitations of a retrospective study, the authors caution that when interpreting the results, one should keep association in mind rather than causation. While we hypothesize that exercise may have a neuroprotective effect in RP based on animal studies, we could not establish a causative relationship in patients with RP from our study. We are, however, cautiously encouraged by our findings that GVF scores trended higher in the active patients, even though they did not reach statistical significance. RP is a devastating disease with no curative treatment. The promising results of exercise [8, 9] and environmental enrichment [27, 28] studies in animals warrant further investigation in humans. As a result, we intend to pursue a prospective, randomized controlled study to investigate the causative effect of exercise on progression of RP. We encourage further investigation of lifestyle modifications such as exercise that may allow patients to have improved quality of life and potentially slow progression of vision loss.

## Figures and Tables

**Table 1:** Demographic and medical history characteristics for 143 subjects with retinitis pigmentosa.

		*n* (%)
Age mean (SD)		46.9 (13.7)
Race^∗^	African American	26 (25%)
	Asian	1 (1.0%)
	Others	2 (2.0%)
	White	73 (72%)
Sex	Female	83 (58%)
	Male	60 (42%)
History of smoking		96 (67%)
Hypertension		42 (29%)
Diabetes		13 (9%)
Cardiovascular disease		9 (6%)

^∗^ *n* = 102; SD: standard deviation.

**Table 2:** Relationship between quality of life survey scores and exercise levels.

		Overall	Active group	Insufficiently active group	*t*-test
		Mean score (SD)	Mean score (SD)	Mean score (SD)	*p* value^∗^
		(*n* = 143)	(*n* = 81)	(*n* = 62)	
PAT-D		26.83 (12.24)	24.35 (8.91)	30.03 (14.99)	0.0096

VFQ-25	Overall	49.77 (19.07)	53.32 (18.85)	45.12 (18.48)	0.0104
	General vision	47.69 (25.33)	50.37 (26.29)	44.19 (23.79)	0.1491
	Ocular pain	85.75 (19.21)	86.88 (19.95)	84.27 (18.24)	
	Near activities	46.50 (26.65)	51.49 (25.22)	39.98 (27.25)	
	Distance activities	40.88 (21.55)	44.86 (22.13)	35.69 (19.77)	
	Social functioning	54.54 (29.20)	55.86 (28.72)	52.82 (29.98)	
	Mental health	46.37 (25.59)	47.69 (25.07)	44.66 (26.37)	
	Role difficulties	57.69 (25.08)	60.34 (26.50)	54.23 (22.85)	
	Dependency	52.68 (30.52)	57.10 (30.45)	46.91 (29.88)	
	Driving	17.07 (28.28)	21.48 (31.40)	11.27 (22.56)	
	Color vision	64.78 (33.84)	73.77 (32.09)	52.87 (32.63)	0.0002
	Peripheral vision	29.79 (26.88)	34.26 (28.63)	23.75 (23.21)	0.0212

SF-36	Physical component Summary	50.45 (10.59)	52.91 (8.84)	47.22 (11.82)	0.0022
	Mental component Summary	51.38 (10.41)	51.11 (9.84)	51.73 (11.20)	0.7313
	Physical functioning	84.11 (22.19)	90.06 (15.19)	76.31 (27.14)	0.0006
	Role physical	75.18 (34.13)	80.00 (30.91)	68.85 (37.26)	
	Role emotional	86.76 (28.42)	87.08 (28.32)	86.34 (28.79)	
	Vitality	60.07 (21.06)	61.19 (20.32)	58.61 (22.08)	
	Mental health	77.84 (18.15)	78.94 (17.37)	76.39 (19.17)	
	Social functioning	85.46 (22.78)	87.50 (20.86)	82.79 (25.02)	0.2367
	Bodily pain	80.14 (22.76)	85.13 (19.23)	73.61 (25.40)	
	General health	67.61 (22.28)	71.08 (21.69)	63.11 (22.40)	0.0365

^∗^ *p* values indicate comparisons between active and insufficiently active subjects. PAT-D: Pepper Assessment Tool for Disability; SD: standard deviation; SF-36: 36-Item Short Form Health Survey; VFQ-25: Visual Function Questionnaire-25.

**Table 3:** Relationship between Goldmann visual field scores and exercise levels.

	Active group	Insufficiently active group	*p* value^∗^
	Mean GVF score (SD)	Mean GVF score (SD)	
Initial measurement^†^	74.78 (70.42) (*n* = 64)	60.07 (70.17) (*n* = 56)	0.2552
Initial measurement^‡^	90.19 (71.94) (*n* = 31)	55.33 (56.29) (*n* = 21)	
Final measurement^‡^	78.65 (62.71) (*n* = 31)	48.71 (47.06) (*n* = 21)	0.0689
Difference (final–initial)^‡^	−11.54 (34.47) (*n* = 31)	−6.62 (50.41) (*n* = 21)	0.6987

^∗^ *p* values indicate statistical comparisons between active and insufficiently active subjects based on the Godin exercise score; ^†^entire group (*n* = 120); ^‡^only includes subjects who completed follow-up testing (*n* = 52); GVF: Goldmann visual field; SD: standard deviation.

**Table 4:** Relationship between Goldmann visual field scores and clinical factors.

	Mean GVF score (SD)	Mean GVF score (SD)	*p* value^∗^
	History of smoking	No history of smoking	
Initial GVF measurement	65.67 (69.14) (*n* = 79)	72.24 (73.43) (*n* = 41)	0.6366
	History of vascular disease	No vascular disease	
Initial GVF measurement	46.21 (45.04) (*n* = 43)	80.04 (78.88) (*n* = 77)	0.0110

^∗^ *p* values indicate comparisons between presence and absence of clinical factors. GVF: Goldmann visual field; SD: standard deviation.

**Table 5:** Relationship between quality of life survey scores and current employment status.

		Overall	Currently employed	Not currently employed	*t*-test
		Mean score (SD)	Mean score (SD)	Mean score (SD)	*p* value^∗^
		(*n* = 143)	(*n* = 66)	(*n* = 76)	
PAT-D		26.83 (12.24)	24.03 (7.84)	29.25 (14.78)	0.0112
VFQ-25	Overall	49.77 (19.07)	56.71 (18.77)	43.78 (17.43)	<0.0001
SF-36	PCS	50.45 (10.59)	52.43 (8.93)	48.79 (1.69)	0.0424
	MCS	51.38 (10.41)	50.90 (9.54)	51.78 (11.25)	0.6208

^∗^ *p* values indicate comparisons between currently employed and not currently employed subjects. MCS: Mental Component Summary of the 36-Item Short Form Health Survey; PAT-D: Pepper Assessment Tool for Disability; PCS: Physical Component Summary of the 36-Item Short Form Health Survey; SD: standard deviation; SF-36: 36-Item Short Form Health Survey; VFQ-25: Visual Function Questionnaire-25.
